# Identification of Inflammatory Response-Related Gene Signature Associated With Immune Status and Prognosis of Lung Adenocarcinoma

**DOI:** 10.3389/fbioe.2021.772206

**Published:** 2021-11-22

**Authors:** Weijie Zou, Li Chen, Wenwen Mao, Su Hu, Yuanqing Liu, Chunhong Hu

**Affiliations:** ^1^ Department of Radiology, The First Affiliated Hospital of Soochow University, Suzhou, China; ^2^ Institute of Medical Imaging of Soochow University, Suzhou, China; ^3^ Department of Interventional Radiology, The First Affiliated Hospital of Soochow University, Suzhou, China

**Keywords:** lung adenocarcinoma, inflammatory response, gene signature, immune status, tumor microenvironment, drug sensitivity

## Abstract

**Background:** Lung adenocarcinoma (LUAD) is an exceedingly diverse disease, making prognostication difficult. Inflammatory responses in the tumor or the tumor microenvironment can alter prognosis in the process of the ongoing cross-talk between the host and the tumor. Nonetheless, Inflammatory response-related genes’ prognostic significance in LUAD, on the other hand, has yet to be determined.

**Materials and Methods:** The clinical data as well as the mRNA expression patterns of LUAD patients were obtained from a public dataset for this investigation. In the TCGA group, a multigene prognostic signature was built utilizing LASSO Cox analysis. Validation was executed on LUAD patients from the GEO cohort. The overall survival (OS) of low- and high-risk cohorts was compared utilizing the Kaplan-Meier analysis. The assessment of independent predictors of OS was carried out utilizing multivariate and univariate Cox analyses. The immune-associated pathway activity and immune cell infiltration score were computed utilizing single-sample gene set enrichment analysis. GO keywords and KEGG pathways were explored utilizing gene set enrichment analysis.

**Results:** LASSO Cox regression analysis was employed to create an inflammatory response-related gene signature model. The high-risk cohort patients exhibited a considerably shorter OS as opposed to those in the low-risk cohort. The prognostic gene signature’s predictive ability was demonstrated using receiver operating characteristic curve analysis. The risk score was found to be an independent predictor of OS using multivariate Cox analysis. The functional analysis illustrated that the immune status and cancer-related pathways for the two-risk cohorts were clearly different. The tumor stage and kind of immune infiltrate were found to be substantially linked with the risk score. Furthermore, the cancer cells’ susceptibility to anti-tumor medication was substantially associated with the prognostic genes expression levels.

**Conclusion:** In LUAD, a new signature made up of 8 inflammatory response-related genes may be utilized to forecast prognosis and influence immunological state. Inhibition of these genes could also be used as a treatment option.

## Introduction

Lung cancer has become the second commonly occurring type of cancer in the world, and it is also the leading contributor of cancer mortality in both men and women, as per the global cancer statistics reported in 2020 (Sung et al., 2021). There are two distinct subtypes of lung cancer that include small cell lung cancer as well as non-small cell lung cancer (NSCLC). The commonly occurring NSCLC is lung adenocarcinoma (LUAD) (Cancer, 2014). The number of LUAD patients is increasing as air pollution and smoking rates decline. Research evidence has suggested that although the prognosis of patients with early LUAD is relatively good, approximately 10–44% of LUAD patients still die within 5 years following the surgical intervention (Goldstraw et al., 2016). However, the 5-year overall survival (OS) rate of patients with advanced LUAD is less than 15% (Kris et al., 2014). Therefore, in addition to standard clinical factors, a new prognostic signature for individualized survival risk assessment must be devised.

The association between inflammation and cancer is well recognized, and its function in the onset and progression of cancer has always been a research topic (Balkwill and Mantovani, 2001; Koliaraki et al., 2020). The inflammation acts as a two-edged sword that can either inhibit or promote tumor development (Greten and Grivennikov, 2019). People can investigate the association between tumor and inflammatory markers by evaluating commonly available measures in the blood. For instance, the Glasgow prognostic score, which includes albumin and C-reactive protein, has independent predictive significance in cancer patients (McMillan, 2013). The clinical systemic inflammation markers such as platelet-lymphocyte ratio (PLR), neutrophil-lymphocyte ratio (NLR), and lymphocyte-monocyte ratio (LMR) were assessed in newly diagnosed lung cancer that has not been previously treated, and these markers portrayed considerable prognostic ability for OS that was independent of formerly identified prognostic factors for lung cancer (Moik et al., 2020). Studies are increasingly supporting the utilization of combined acute-phase proteins to create an inclusive predictive score for cancer premised on inflammation. Some inflammatory response-related genes, in addition to serum indicators, are utilized to predict tumor prognosis and metastatic potential (Budhu et al., 2006; Lin et al., 2021). Current research on the association between inflammation-related genes and LUAD is limited.

LUAD patients’ clinical information and mRNA expression profiles were collected from a public database for this investigation. Then, using differentially expressed genes (DEGs) that were linked to an inflammatory response from The Cancer Genome Atlas (TCGA) cohort, we built a prognostic signature and confirmed the reliability and stability of the model utilizing the Gene Expression Omnibus (GEO) cohort. To investigate its potential mechanism, we employed functional enrichment analysis. Moreover, we also looked at the link between the types of immune infiltrates and the expression of prognostic genes. Finally, we searched into the link between the expression of prognostic genes, cancer chemoresistance, as well as tumor stemness.

## Materials and Methods

### Data Collection (TCGA-LUAD Cohort and GEO(GSE68465) Cohort)

The TCGA (https://www.cancer.gov/tcga) (*n* = 594) and GEO (http://www.ncbi.nlm.nih.gov/geo/) (*n* = 442) databases were used to obtain clinical data and RNA sequencing data. Patients who did not have a survival status or whose follow-up duration was less than a day were omitted from the study. Both TCGA and GEO made their data available to the public, in accordance with their respective publication requirements and data access policies. Moreover, in the Molecular Signatures database, 200 inflammatory response-related genes were discovered (Supplementary Table S1).

### Construction and Validation of a Prognostic Inflammatory Response-Related Gene Signature

In the TCGA cohort, DEGs between non-tumor as well as tumor tissues were detected utilizing the “limma” R package with a false discovery rate <0.05 and a fold change >2. The inflammatory response-related genes with prognostic significance were evaluated utilizing Univariate Cox analysis. To reduce the overfitting risk, LASSO-penalized Cox regression analysis was utilized to build a prognostic model (Simon et al., 2011). With the “glmnet” R package, the LASSO algorithm was utilized to choose and compress variables so that some regression coefficients were stringently equivalent to 0, resulting in a model that was interpretable. The prognostic model’s penalty parameter (λ) was assessed utilizing tenfold cross-validation, and we adhered to the minimum requirements. The expression levels of each inflammatory response-related gene, as well as the matching regression coefficient, were utilized to identify the patient’s risk scores. The algorithm was defined as score = e^sum (each gene’s expression × corresponding coefficient)^. The participants were categorized into low- and high-risk cohorts premised on their median risk score. To investigate the distribution of distinct cohorts with reference to gene expression levels in the built model, t-SNE and PCA analyses were done using the “Rtsne” and “ggplot2” R packages. The “survminer” R package was utilized to undertake a survival analysis on the OS of low- and high-risk cohorts. The time-dependent ROC curve analysis so as to assess the prognostic signature’s predictive ability. In addition, multivariate and univariate Cox analyses were employed to investigate the signature’s independent prognostic value.

### Functional Enrichment Analysis

Gene Set Enrichment Analysis (GSEA) software 4.1 was employed to perform Gene Ontology (GO) and Kyoto Encyclopedia of Genes and Genomes (KEGG) analyses based on the DEGs between the low- and high-risk cohorts using GSEA. Single-sample GSEA (ssGSEA) with the “GSVA” R package was used to compare the activity of 13 immune-related pathways and the infiltration scores of 16 immune cells between the low- and high-risk cohorts.

### Immune Response Analysis and Tumor Microenvironment

Stromal cells and immune cells infiltration levels in distinct tumor tissues were assessed utilizing stromal and immune scores (Yoshihara et al., 2013). The link between the risk score and the stromal/immune scores was verified using the Spearman correlation. A two-way ANOVA analysis was used to see if there was a connection between the subtype of immune infiltration and risk score. To quantify tumor stem cell-like traits, researchers analyzed data collected from the epigenetics and transcriptome of TCGA tumor samples (Dib et al., 2017). The relationship between risk score and tumor stemness was investigated using the Spearman correlation test.

### Analysis of Sensitivity to Chemotherapy

The CellMiner interface (https://discover.nci.nih.gov/cellminer) was utilized to access the NCI-60 dataset, which included 60 distinct cancer cell lines from 9 distinct kinds of malignancies. The link between medication sensitivity and prognostic gene expression was investigated utilizing Pearson correlation analysis. A correlation analysis was performed to investigate the effectiveness of 263 medications authorized by the FDA or currently in clinical studies (Supplementary Table S2).

### Statistical Analysis

The DEGs between tumor samples and surrounding tissues were assessed utilizing the WilCoxon test. Subsequently, the Chi-squared test was performed for comparison of the various proportions. Next, ssGSEA scores of immunological pathways or immune cells were compared between low- and high-risk cohorts utilizing the Mann-Whitney, and the adjustment of the *p*-value was done utilizing the Benjamini and Hochberg technique. The Kaplan-Meier method was employed for the comparison of the differences in OS among distinct cohorts. To screen the independent determinants of OS, multivariate and univariate Cox analyses were done. Spearman or Pearson correlation analysis was utilized to examine the connection between prognostic gene expression level or prognostic model risk score and stemness score, drug sensitivity, immune score, and stromal score, and. Plots were made using R software (Version 3.6.3) and the tools survminer, corrplot, venn, ggplot2, pheatmap, igraph, and ggpubr. A two-tailed *p* < 0.05 depicted statistical significance in all statistical results.

## Results

**FIGURE 1:**
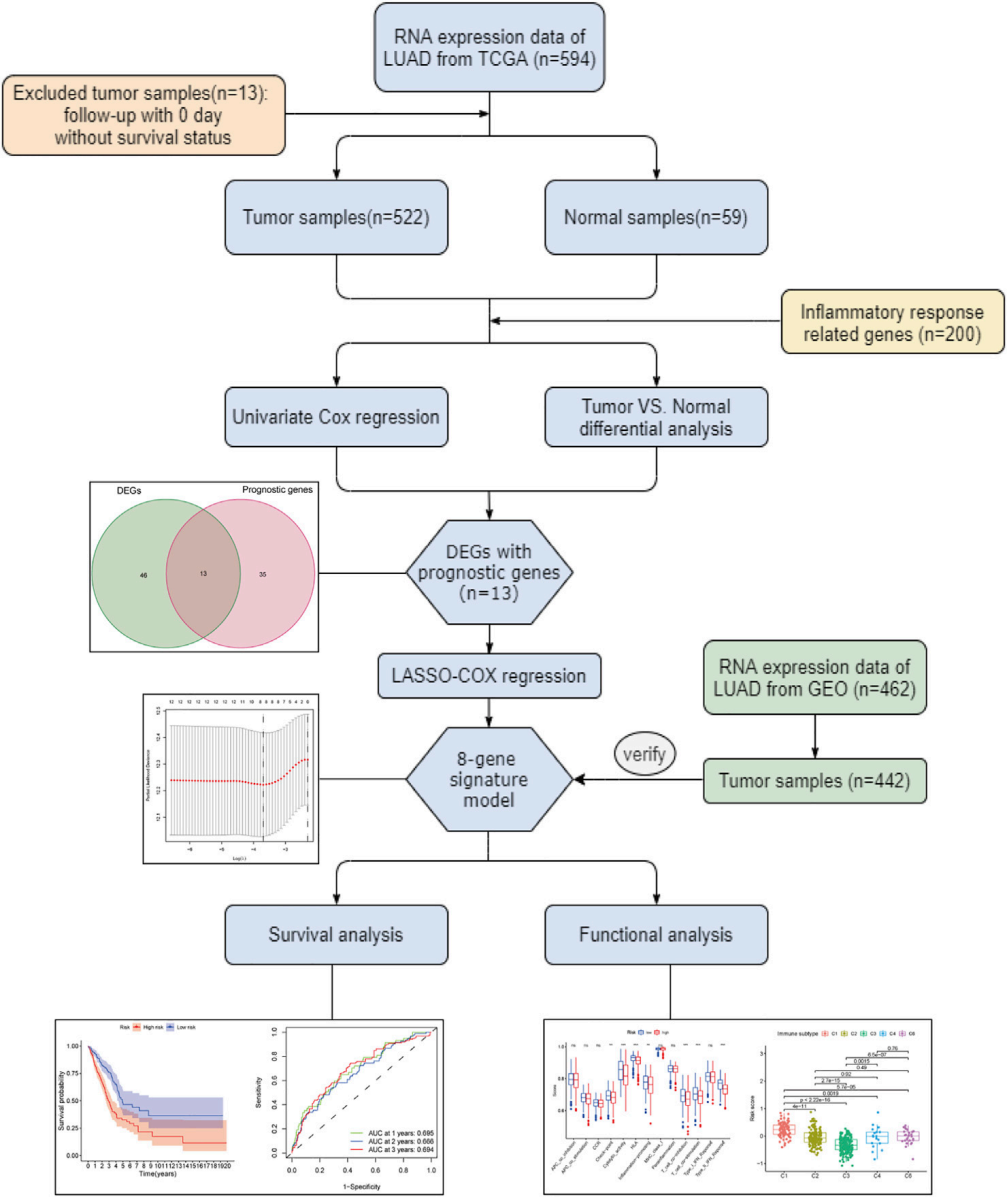
Flow chart of the whole study.

Figure 1 depicts this study’s flowchart. The research comprised 522 LUAD patients from the TCGA-LUAD group and 442 LUAD participants from the GEO (GSE68465) group. The specific clinical characteristics of these individuals were presented in Table 1.

**TABLE 1:** Clinical characteristics of the LUAD patients used in this study.

	TCGA cohort	GEO cohort
No. of patients	522	442
Age (median, range)	66 (33–88)	65 (33–87)
Gender		
Female	280 (53.6%)	219 (49.5%)
Male	242 (46.4%)	223 (50.5%)
Stage		
I	279 (53.4%)	115 (26%)
II	124 (23.8%)	257 (58.1%)
III	85 (16.3%)	70 (15.9%)
IV	26 (5.0%)	0 (0%)
Unknown	8 (1.5%)	0 (0%)
Survival status		
Alive	334 (64%)	207 (46.8%)
Deceased	188 (36%)	235 (53.2%)

### Identification of Prognostic Inflammation-Related DEGs in the TCGA Cohort

**FIGURE 2:**
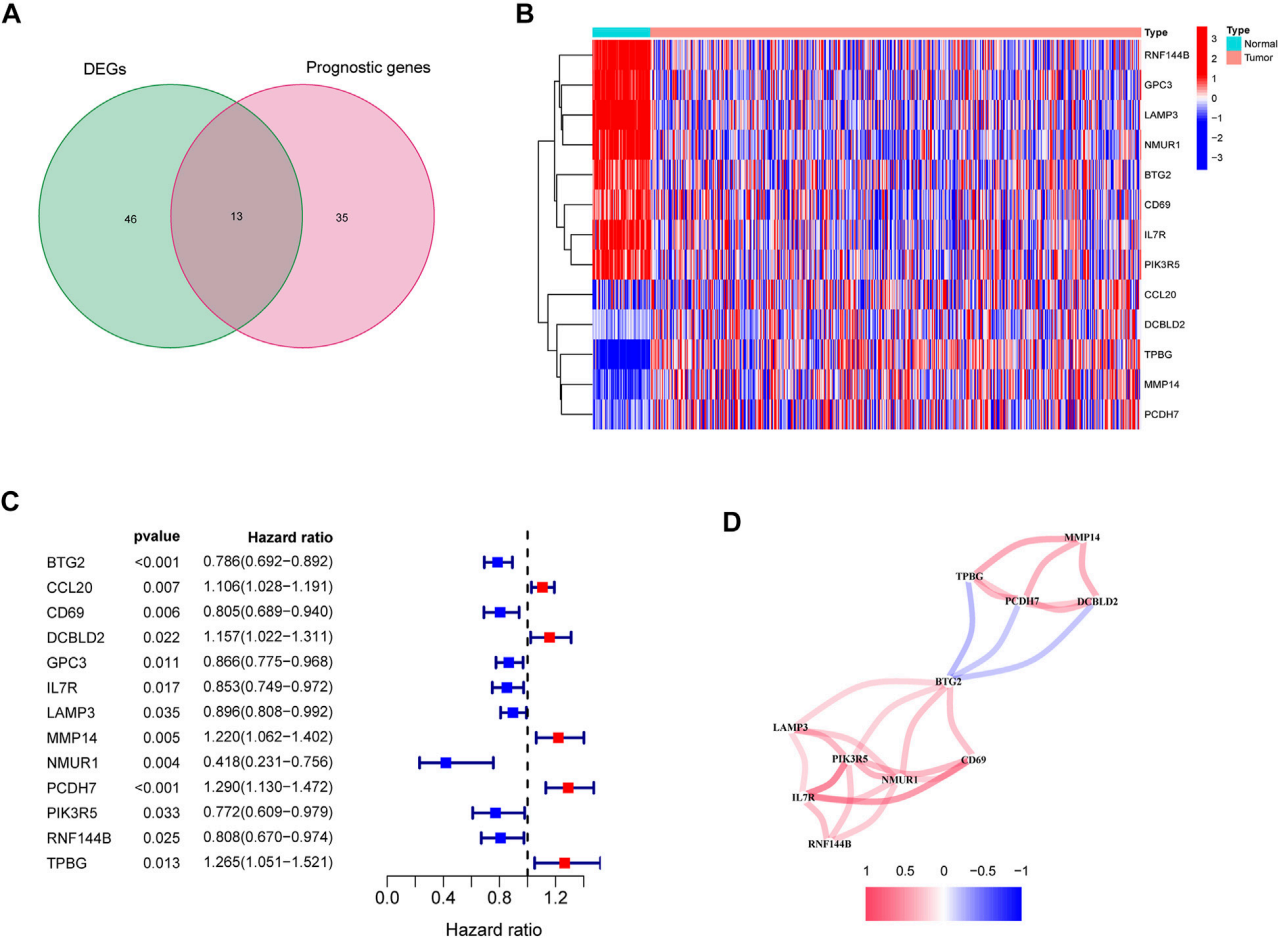
Identification of the candidate inflammatory response-related genes in the TCGA cohort. **(A)** Venn diagram to identify DEGs between LUAD tissues and adjacent normal tissues. **(B)** The 13 overlapping genes expression between LUAD tissues and adjacent normal tissues. **(C)** Forest plots showing the results of the association between 13 overlapping gene expression and OS. **(D)** The correlation network of candidate genes.

In the tumor tissues as well as the adjacent non-tumor tissues, 59 inflammatory response-related genes were found to be differently expressed. Based on the outcomes of the univariate Cox analysis, 13 of them were associated with OS (Figures 2A,B). The prognostic indicators consisted of 13 inflammatory response-related genes, and the PCDH7 gene risk ratio was 1.29 (95% CI = 1.130–1.472, *p* < 0.001, Figure 2C). Figure 2D depicts the relationship among these genes.

### Designing a Prognostic Model in the TCGA Group

**FIGURE 3:**
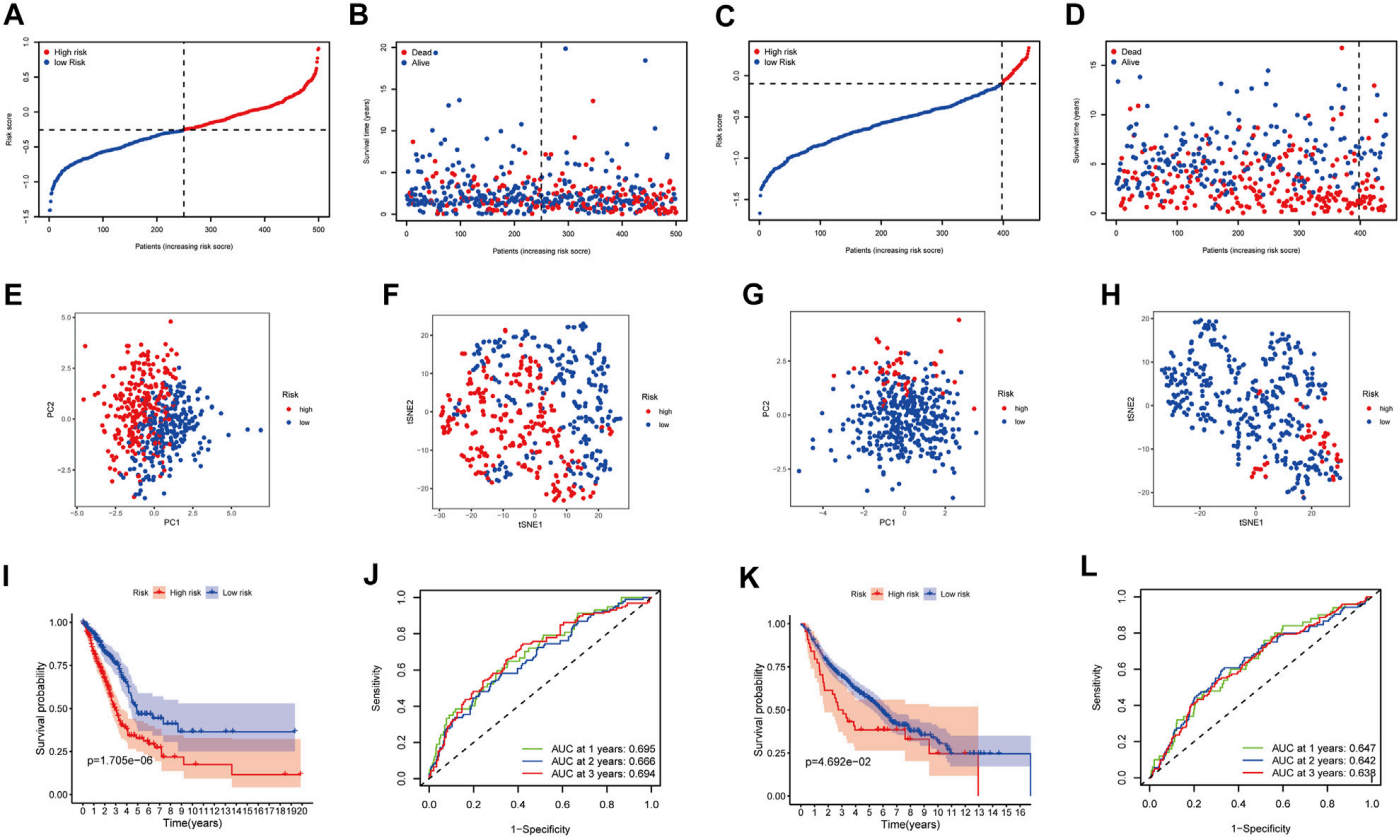
Prognostic analysis of the 8-gene signature model in the TCGA cohort and GEO cohort. TCGA cohort **(A,B,E,F,I,J)**, GEO cohort **(C,D,G,H,K,L)**. **(A,C)** The median value and distribution of the risk scores. **(B,D)** The distribution of OS status. **(E,G)** PCA plot. **(F,H)** t-SNE analysis. **(I,K)** Kaplan-Meier curves for OS of patients in the high- and low-risk groups. **(J,L)** AUC time-dependent ROC curves for OS.

The LASSO-Cox regression was utilized to evaluate the expression profiles of the aforementioned 13 genes, which was ensued by the development of a prognostic model. Premised on the ideal value of λ, an 8-gene marker was identified (Supplementary Figure S1). The risk score was calculated as follows: e ^(expression level of BTG2 * −0.088+expression level of CCL20 * 0.083+expression level of CD69 * −0.044+expression level of GPC3 * −0.012+expression level of IL7R *−0.119+expression level of MMP14 * 0.068+expression level of NMUR1 * −0.090+expression level of PCDH7 * 0.148)^. As per the median cut-off value, patients were separated into two cohorts (Figure 3A). The high-risk category in the TCGA cohort was determined to be strongly related to the more advanced TNM stage (Table 2). The patients in distinct risk categories were dispersed in two ways, according to PCA and t-SNE analysis (Figures 3E,F). Furthermore, the scatter chart revealed that high-risk patients had an increased likelihood of dying earlier as opposed to low-risk ones (Figure 3B). The Kaplan-Meier curve constantly demonstrated that high-risk patients had a considerably shorter OS contrasted with those with low risk (Figure 3I, *p* < 0.001). For the investigation of the prognostic model’s survival prediction, time-dependent ROC curves were created, with the area under the curve (AUC) ranging between 0.695, 0.666, and 0.694 at 1-, 2-, and 3 years in that order (Figure 3J). Survival analysis was carried out premised on the optimal cut-off expression value for each of the prognostic genes to investigate the relationship between the prognostic genes and prognosis, which revealed that an elevated expression of these genes was all considerably linked to poor OS except GPC3 (Supplementary Figures S2A–H, *p* < 0.05).

**TABLE 2:** Baseline characteristics of the patients in different risk groups.

Characteristics	TCGA cohort				GEO cohort		
	High risk	Low risk	*p* value		High risk	Low risk	*p* value
Age							
≤65	136 (54.4%)	101 (40.4%)	0.0021		21 (47.73%)	210 (52.76%)	0.6343
>65	109 (43.6%)	144 (57.6%)			23 (52.27%)	188 (47.24%)	
unknow	5 (2%)	5 (2%)			—	—	
Gender							
Female	127 (50.8%)	143 (57.2%)	0.1783		20 (45.45%)	199 (50%)	0.6793
Male	123 (49.2%)	107 (42.8%)			24 (54.55%)	199 (50%)	
Stage							
I-II	182 (72.8%)	205 (82%)	0.0095		32 (72.73%)	353 (88.69%)	0.0058
III-IV	65 (26%)	40 (16%)			12 (27.27%)	45 (11.31%)	
unknow	3 (1.2%)	5 (2%)			—	—	

### Validation of the 8-Gene Signature in the GEO Cohort

The participants in the GEO group were also grouped into low-risk or high-risk cohorts premised on the median value from the TCGA group to assess the stability of the model established from the TCGA group (Figure 3C). t-SNE and PCA analysis indicated a distinct dispersal of patients in the two groupings, similar to the TCGA cohort results (Figures 3G,H). Correspondingly, as contrasted with the low-risk cohort, patients in the high-risk cohort had an increased likelihood of dying prematurely (Figure 3D) and a lower survival period (Figure 3K). Furthermore, the AUC of the eight gene signature was 0.647, 0.642, and 0.638 at 1-, 2-, and 3-year in that order (Figure 3L).

### Independent Prognostic Value of the 8-Gene Signature

**FIGURE 4:**
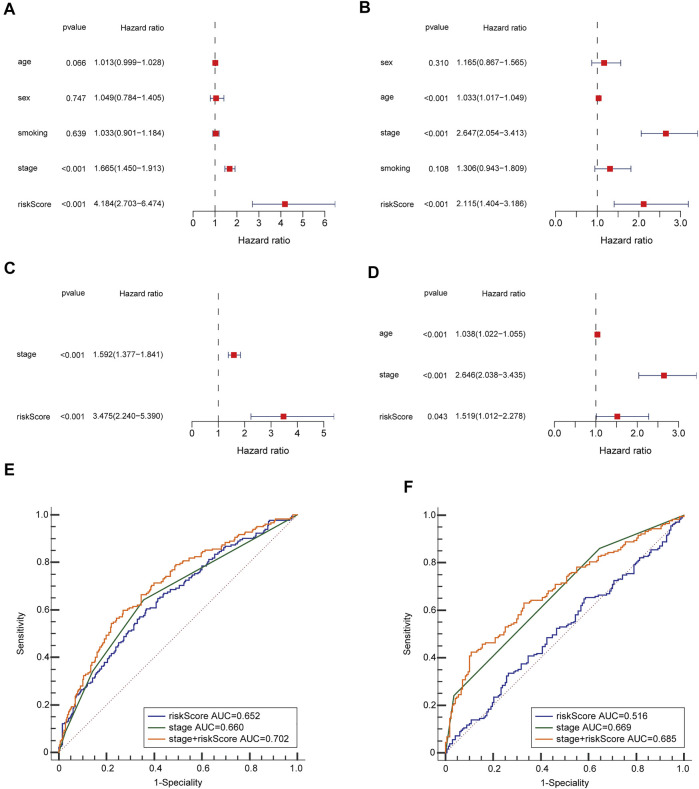
OS-related factors were screened, and the prognostic accuracy of risk score and clinicopathological factors were compared. TCGA cohort **(A,C,E)**, GEO cohort **(B,D,F)**. **(A,B)** OS-related factors were screened by Univariate Cox regression analyses. **(C,D)** OS-related factors were screened by Multivariate Cox regression analysis. **(E,F)** Time-dependent ROC curve was used to compare the prognostic accuracy of risk score, tumor stage, and the combination of risk score. and tumor stage in 3-year.

To see if the risk score could be an independent predictive factor for OS, we used both multivariate and univariate Cox analysis of factors. The univariate Cox analysis illustrated that the risk scores in the GEO and TCGA groups had a significant correlation with OS (GEO group: HR = 2.115, 95% CI = 1.404–3.186, *p* < 0.001; TCGA group: HR = 4.184, 95% CI = 2.703–6.474, *p* < 0.001) (Figures 4A,B). Multivariate Cox analysis revealed that the risk score remained to be an independent predictor of OS after accounting for other confounding factors (TCGA group: HR = 3.475, 95% CI = 2.240–5.390, *p* < 0.001; GEO group: HR = 1.519, 95% CI = 1.012–2.278, *p* = 0.043) (Figures 4C,D). The ROC curve research revealed that the risk score exhibited a better prognostic predictive precision and that when paired with the tumor stage, it offered a highly precise 3-year OS forecast in LUAD patients, regardless of whether they were in the TCGA (AUC = 0.702) or GEO dataset (AUC = 0.685) (Figures 4E,F). As a result, LUAD’s predictive value was outstanding when the risk score and clinicopathological parameters were combined.

### Prognostic Model Risk Score and Clinical Features

**FIGURE 5:**
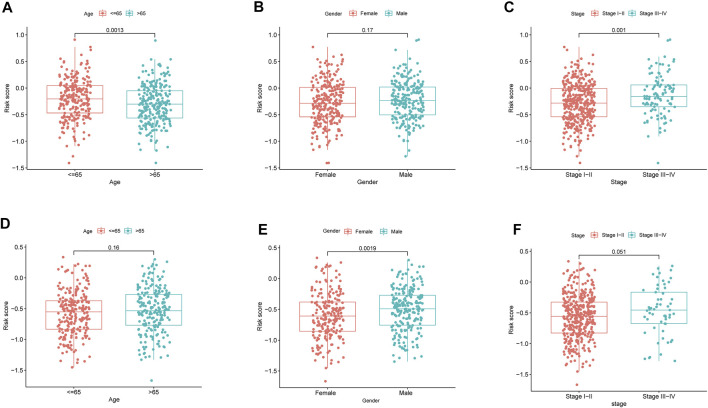
The risk score in different groups divided by clinical characteristics. TCGA cohort **(A–C)**, GEO cohort **(E–F)**. **(A,D)** Age. **(B,E)** Gender. **(C,F)** Tumor stage.

By analyzing the association of risk score with the clinical characteristics of LUAD patients, we showed that the risk score was significantly higher in patients’ age ≤65 (*p* = 0.0013) compared with >65 in TCGA cohort, while the risk score was no significant difference between the ages ≤65 and >65 years in GEO cohort (*p* = 0.16) (Figures 5A,D). The risk score was significantly higher in male (*p* = 0.0013) compared with female in GEO cohort, while there was no significant difference in TCGA cohort (*p* = 0.0019) (Figures 5B,E). Our findings depicted risk scores that were considerably greater in tumor stage III-IV as opposed to tumor stage I-II in both the GEO and TCGA datasets when we investigated the relationship between risk scores and clinical characteristics of patients with LUAD (GEO: *p* = 0.051; TCGA: *p* = 0.001) (Figures 5C,F). Furthermore, the expression of BTG2, IL7R, and NMUR1 was dissimilar between age ≤65 years and age >65 years, and the level of expression of CD69, GPC3, and IL7R was distinct between males and females (*p* < 0.05, Supplementary Figures S3A,B). In addition, the expression of BTG2, CD69, GPC3, and IL7R were considerably elevated in tumor stages III-IV as opposed to tumor stage I-II (*p* < 0.05, Supplementary Figure S3C).

### Immune Status and TME Analysis

**FIGURE 6:**
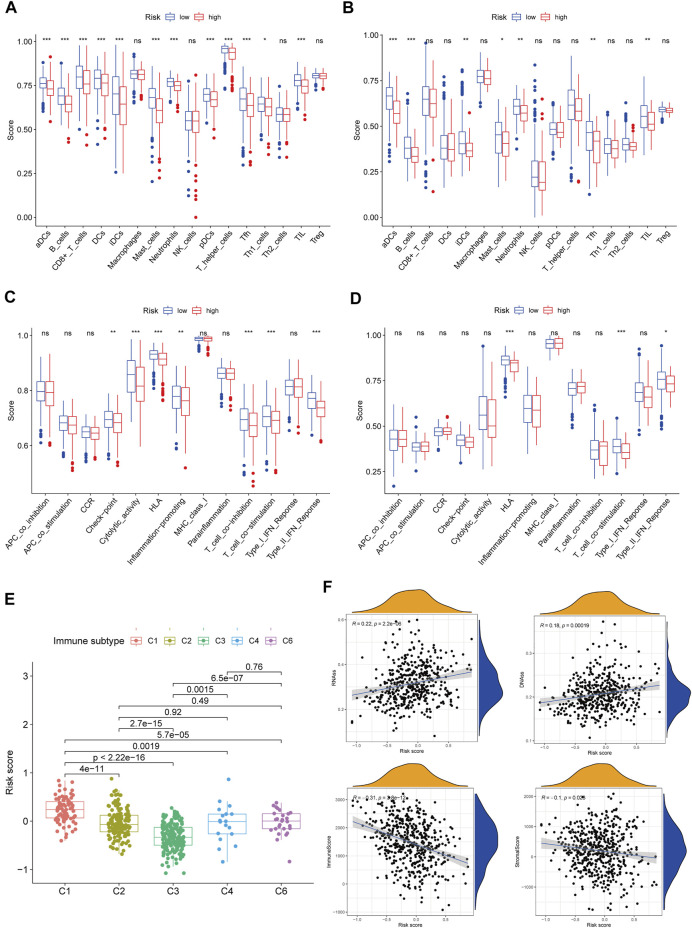
Immune status between different risk groups and the association between risk score and tumor microenvironment. TCGA cohort **(A,C)**, GEO cohort **(B,D)**. **(A,B)** The scores of 16 immune cells and **(C,D)** 13 immune-related functions were showed in boxplots. **(E)** Comparison of the risk score in different immune infiltration subtypes. **(F)** The relationship between risk score and RNAss, DNAss, Stromal Score and Immune Score. *p* values were showed as: ns, not significant; *p < 0.05; **p < 0.01; ***p < 0.001.

ssGSEA was employed in quantifying the enrichment scores of various immune cell subpopulations, related pathways, and functions in order to further assess the connection between immune status and risk score. The antigen presentation process, involving aDCs, DCs, iDCs, pDCs, and HLA, was shown to be considerably higher in the low-risk cohort in the TCGA class (*p* < 0.05, Figures 6A,C). The fractions of T helper cells, CD8^+^ T cells, Th1 cells, Tfh cells, TIL, T cell co-inhibition, and T cell co-stimulation in the low-risk cohort were greater than in the high-risk cohort, showing variances in T cell regulation between the two cohorts. In addition, the high-risk cohort had greater scores for B cells, mast cells, neutrophils, inflammation-promoting, check-point, type II IFN response activity, and cytolytic activity (*p* < 0.05). Comparing the two risk subcategories in the GEO group yielded results that were similar to those in the TCGA (*p* < 0.05, Figures 6B,D).

We investigated the link between immune infiltrates and risk scores to find out more about the link between risk scores and immune components. In human tumors, six types of immune infiltrate were established that ranges from tumor-promotion to tumor-suppression (Tamborero et al., 2018), including C1 (wound healing), C2 (INF-γ dominant), C3 (inflammatory), C4 (lymphocyte depleted), C5 (immunologically quiet), and C6 (TGF-β dominant). Because none of the patient samples in LUAD corresponded to the C5 immune subtype, C5 immune subtypes were excluded from the study. The immune infiltration of LUAD in the TCGA dataset was evaluated and contrasted with a risk score, with the outcomes revealing that an elevated risk score was considerably linked to C1, whereas a reduced risk score was considerably linked to C3 (Figure 6E). High expression of BTG2, CD69, GPC3, IL7R, MMP14, and NMUR1 were significantly related with C3, and high expression of CCL20, MMP14, and PCDH7 was clearly connected with C1, as shown in Supplementary Figure S4.

**FIGURE 7:**
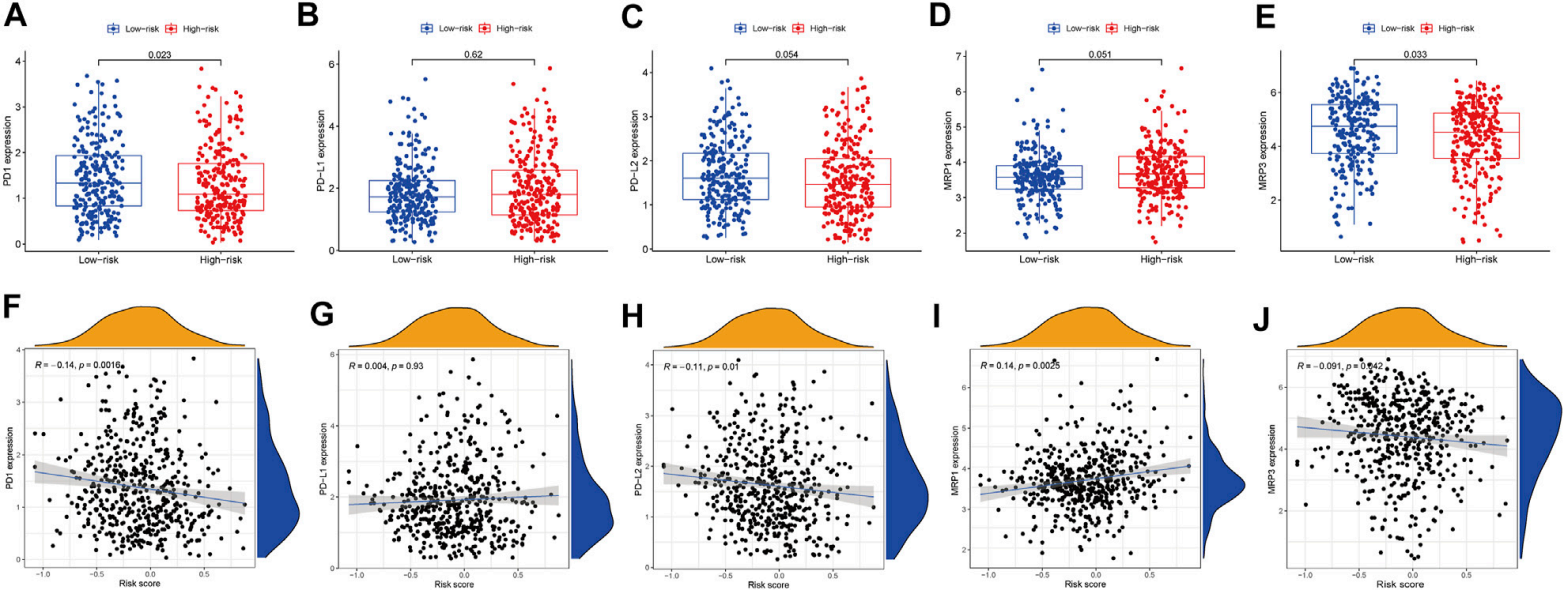
The comparison of the expression levels of PD-1, PD-L1, PD-L2, MRP1 and MRP3 between different risk groups and correlation analysis between risk score and the expression levels of PD-1, PD-L1, PD-L2, MRP1 and MRP3. **(A,F)** PD-1. **(B,G)** PD-L1. **(C,H)** PD-L2. **(D,I)** MRP1. **(E,J)** MRP3.

In cancer immune evasion, the PD-1/PD-L2 and PD-1/PD-L1 pathways are important regulators. Immune checkpoints’s expression such as PD-L2 and PD-L1 are essential markers for personalized treatment. In the cohort categorized as high-risk, the PD-1 and PD-L2 expression levels were distinct from those in the low-risk cohort (Figures 7A,C) and revealed a negative relationship with the risk score (Figures 7F,H). The PD-L1 expression levels and immunological checkpoints were not substantially related to the low- and high-risk cohorts (Figures 7B,G). With regards to tumor medication resistance genes, patients in the high-risk cohort had elevated MRP1 expression as opposed to low-risk patients, which was positively connected with risk scores, but MRP3 was the inverse. With regards to tumor drug resistance genes, MRP1 expression was elevated in the high-risk cohort as opposed to the low-risk cohort and had a positive correlation with risk scores, whereas MRP3 was the opposite (Figures 7D,E,I,J).

The RNA stemness (RNAss) score, which is premised on mRNA expression, and the DNA stemness (DNAss) score, which is premised on DNA methylation pattern, can both be used to determine tumor stemness (Malta et al., 2018). The tumor immune microenvironment was estimated using stromal and immune scores. The goal of the correlation analysis was to see if the risk score was linked to the immune microenvironment and tumor stem cells. The outcomes illustrated that the risk score was significantly and positively linked to RNAss and DNAss but significant and negatively linked to immune and stromal score (*p* < 0.05) (Figure 6F).

### Pathway Analyses and Biological Function

**FIGURE 8:**
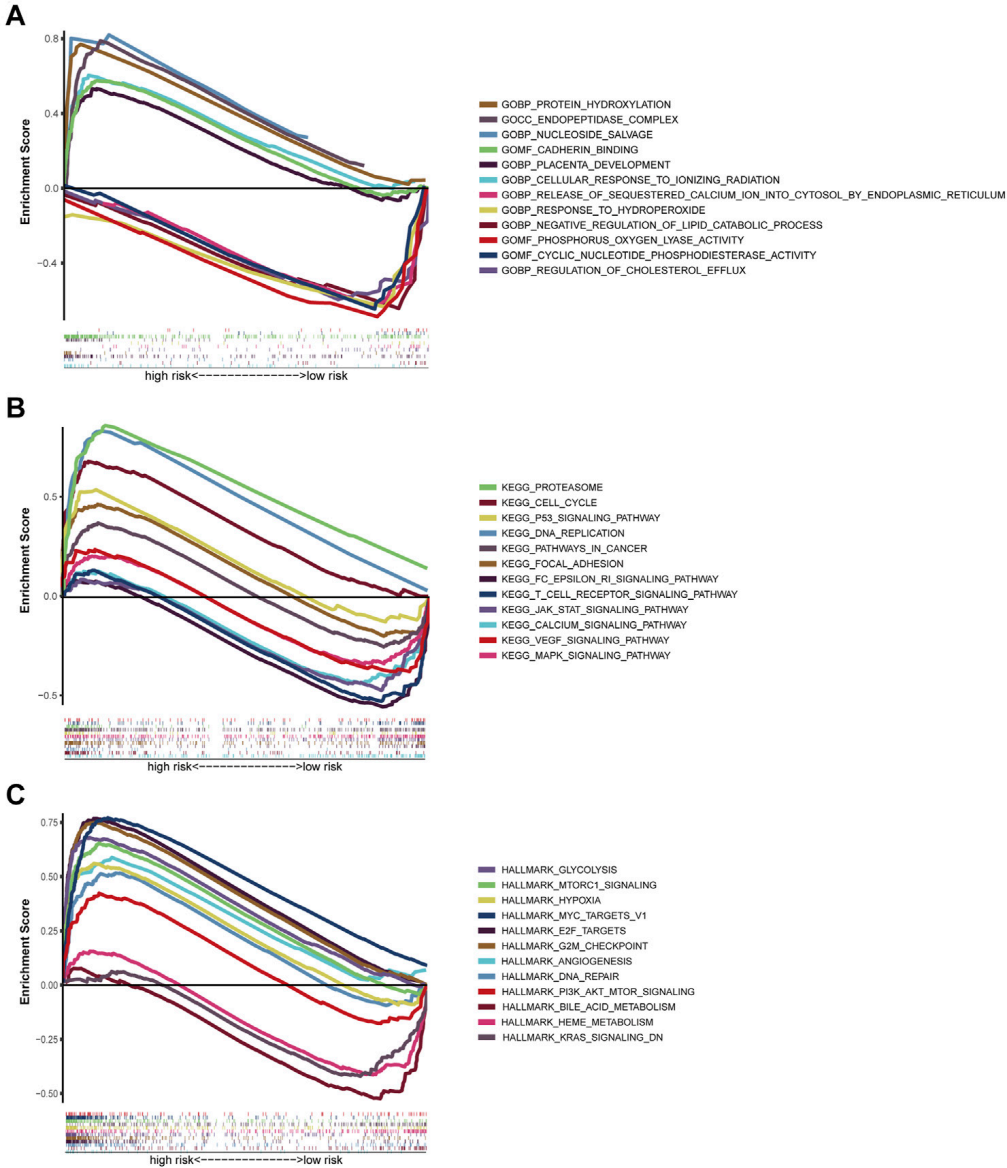
Gene set enrichment analysis of Biological functions and pathways. **(A)** GO, Gene Ontology. **(B)** KEGG, Kyoto Encyclopedia of Genes and Genomes. **(C)** Hallmark gene set.

The GSEA was employed for comparison between the low- and high-risk cohorts in terms of GO function and KEGG pathway enrichment. Cell cycle phase transition regulation was considerably enriched in the high-risk cohort, while intracellular activity was considerably enriched in the low-risk cohort, according to GO function enrichment analysis (Figure 8A). Also, enrichment of 12 KEGG pathways took place in the high- and low-risk cohorts with a *p* < 0.05 (Figure 8B). Some cancer-related pathways such as Cell Cycle, Proteasome, and P53, were found to be enriched in the high-risk cohort, while JAK-STAT, MAPK, and VEGF were revealed to be enriched in the low-risk cohort. In addition, FcεRI receptor, calcium, and T cell receptor were also revealed in the KEGG pathways, which were associated with inflammatory responses. PI3K-AKT-mTOR-Signaling, mTORC1-Signaling G2/M checkpoint, hypoxia, and epithelial-mesenchymal transition pathways were statistically significant programs, according to GSEA utilizing TCGA data from the Hallmarks gene sets (Figure 8C).

### Expression of Prognostic Genes and Sensitivity of Cancer Cells to Drugs

**FIGURE 9:**
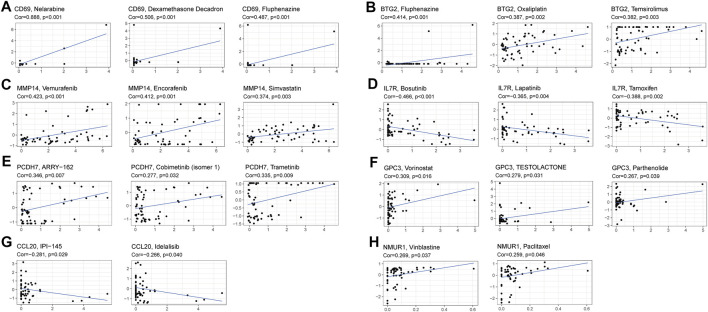
Scatter plot of relationship between prognostic gene expression and drug sensitivity. **(A)** CD69. **(B)** BTG2. **(C)** MMP14. **(D)** IL7R. **(E)** PCDH7. **(F)** GPC3. **(G)** CCL20. **(H)** NMUR1.

The prognostic genes expression in NCI-60 cell lines was explored, as well as the association between their levels of expression and medication sensitivity. The findings revealed that all prognostic genes were linked to some chemotherapy drug sensitivity (*p* < 0.05) (Figure 9). For instance, higher expression of CD69, BTG2, MMP14, PCDH7, GPC3, and NMUR1 has been related to greater cancer cell drug sensitivity to a variety of chemotherapeutic agents, including oxaliplatin, vemurafenib, trametinib, paclitaxel, and vinblastine, etc. In contrast, elevated expression of IL7R and CCL20 was linked to greater cancer cell drug resistance to bosutinib, lapatinib, tamoxifen, IPI−145, and idelalisib.

## Discussion

The treatment of LUAD has advanced dramatically as a result of the development of sequencing technology as well as the dawn of a period of precision medicine, but lung cancer remains the most fatal cancer on a global scale. We can’t always diagnose and forecast the therapeutic effects of LUAD because there are few reliable indicators. Therefore, developing a novel approach for reliably identifying LUAD is critical for the disease’s diagnosis and prognosis. Accompaniment fragments, circulating blood protein profiling, DNA methylation, tumor DNA, miRNAs (Seijo et al., 2019), circulating tumor cells (Maly et al., 2019), and a plasma miRNA panel (Wadowska et al., 2020) have all been shown to have high accuracy in LUAD prognosis in previous research. Moreover, inflammatory response-related serum biomarkers including PLR, LMR, NLR, and SII (systemic immune-inflammation index) also portray better performance in forecasting the prognosis of LUAD (Nost et al., 2021). Though, there hasn’t been any research on the inflammatory response-related gene signature as a predictive predictor for LUAD. Studies have shown that gene signatures that are immune-related (Yi et al., 2021), hypoxia-related (Sun et al., 2020), ferroptosis-related (Zhang et al., 2021), and energy metabolism-related (Zhang et al., 2019) forecast 3-year OS in a similar way as the findings in this research. Besides the excellent predictive performance of LUAD, the inflammatory response-related gene signature established in this research has greater advantages as opposed to the gene signature aforementioned. For instance, it can differentiate tumor resistance genes and immune checkpoint genes into low- and high-expression categories, and it has been demonstrated that risk scores are connected to resistance to several chemotherapeutic medicines. High-throughput sequencing was used in our investigation to evaluate the prognostic signature genes expression levels, which is a common technology that can produce accurate results.

For this research, we explored the expression of 200 inflammatory response-related genes in LUAD tissues as well as how they relate to OS. From the TCGA cohort, 59 DEGs were selected. Univariate Cox analysis depicted that 13 of the DEGs were related to OS. Subsequently, a prognostic model was created utilizing the LASSO regression analysis. The model included 8 inflammatory response-related genes and was verified in the GEO group. The participants were grouped into low- and high-risk cohorts premised on their median risk score. The outcomes illustrated that the high-risk cohort was linked to the shorter OS and higher tumor grade. Independent prognostic analysis illustrated that risk score was an independent predictor for OS.

This research created a prognostic model comprised of 8 inflammatory response-related genes. CCL20, MMP14, and PCDH7 were upregulated in LUAD tumor tissues and linked to poor clinical outcomes, while BTG2, CD69, GPC3, IL7R, and NMUR1 are the opposite. CCL20 is one of the important members of chemokine family, which was reported that may be a protective factor or prognostic risk factor for LUAD (Bao et al., 2016). CCL20-encoded proteins are capable of chemotaxis of lymphocytes, allowing the tumor to form an immune tolerance state (Schutyser et al., 2003). In patients with NSCLC, the CCL20 gene and protein are overexpressed, and autocrine of CCL20 can promote the migration and proliferation of lung cancer cells (Mao et al., 2021). MMP14 is a member of the matrix metalloproteinase family and contributes to a key function in cancer metastasis, its expression is significantly correlated with poor OS (Stawowczyk et al., 2017; Infante et al., 2018; Zhang et al., 2020). PCDH7 is a member of the cadherin superfamily and has been found to promote the metastasis of lung cancer cells (Chen et al., 2016). BTG2 is a recently recognized tumor suppressor belonging to the TOB/BTG family, and a study shows that BTG2 expression is reduced in NSCLC tissues and is linked to shorter OS for patients (Chen et al., 2020). Kim et al. (2003) demonstrated GPC3 to be a candidate gene for lung tumor suppression. Ning et al. (2021) found GPC3 expression was significantly correlated with gender and tumour stage in LUAD samples. IL7R has been explored as aggressive tumor features for patients with LUAD: KRAS mutation, larger tumor size, lymphovascular invasion, high-grade morphology, and more frequent recurrence (Suzuki et al., 2013). NMUR1 is a receptor for the neuropeptide NMU, and NMUR1 signaling promotes inflammatory ILC2 responses, highlighting the importance of neuro-immune crosstalk in allergic inflammation at mucosal surfaces (Wallrapp et al., 2017).

We investigated the function of the risk score in immune infiltration type to acquire a comprehensive understanding of the interplay between immune components and risk score. We found that high-risk score was closely linked to C1, whereas low-risk score was strongly linked to C3, implied that C1 stimulates tumor occurrence and progression while C3 was a good protective factor. Because highly cytotoxic immunophenotypes can limit the occurrence and progression of tumors, this study was consistent with the findings of prior investigations (Tamborero et al., 2018). With regard to the relationship between clinical features and risk score, a high-risk score was found to be strongly connected with tumor stages III-IV, implying that a high-risk score is unquestionably linked to a poor prognosis.

However, because there has been little research on these genes, it is unclear if they influence LUAD patients’ prognosis through an inflammatory response. Tumor-related signal pathways like MAPK, p53, and JAK-STAT were considerably enriched in the GSEA analysis, and incessant activation of these pathways is connected to LUAD, which could be new treatment targets (Chou et al., 2019; Mohrherr et al., 2019; Stutvoet et al., 2019). Inflammation-related signal pathways including Calcium, T cell receptor, and FcεRI receptor pathways were significantly enriched, indicating that the inflammatory response is associated with tumor progression. Besides, the low-risk cohort exhibited higher fractions of mast cells, Dcs, and neutrophils. Existing evidence has illustrated that the presence of tumor-associated t mast cells, DCs, and some neutrophils might have a protective influence on the progression of the tumor, they were thought to be beneficial for survival in NSCLC (Welsh et al., 2005; Engblom et al., 2017; Li et al., 2017). We show that anti-PD-L2 antibodies were increased in the low-risk group, this finding was aligned with the outcomes of former studies (Shinchi et al., 2019). In our research, the low-risk cohort had a greater immune checkpoint score as opposed to the high-risk cohort, and the risk score was negatively connected with PD-1 and PD-L2 expression. Hence, the created prognostic model has the potential to guide treatment decisions by predicting immune checkpoint expression levels. Furthermore, a high-risk score was linked to reductions in the type II IFN response activity, which is critical for promoting tumor elimination, promoting anti-tumor immunity, as well as tumor immune surveillance (Shankaran et al., 2001; Street et al., 2001; Wang et al., 2015). Finally, higher T helper cell, CD8^+^ T cell, TIL, Tfh cell, Th1 cell, T cell co-inhibition, and T cell co-stimulation activities in the low-risk cohort implied that the immune regulatory role in the high-risk cohort has been inhibited, and this may be the main reason behind its poor prognosis.

Currently, cancer biology is shifting from a “cancer cell-centered” perspective to one whereby the cancer cells are embedded in a stromal cells network that includes inflammatory immune cells, vascular cells, and fibroblasts. The TME is comprised of these cells (Greten and Grivennikov, 2019). Cancer stem cell-like cells (CSCs) could be formed from a variety of sources such as progenitor cells, long-lived stem cells, and dedifferentiation of non-stem cells (Malta et al., 2018). Because of their propensity to undergo self-renewal and invasion, CSCs promote tumor growth. This is why treatment-induced drug resistance is a problem for these cells (Huang et al., 2010). The risk score was substantially linked to RNAss, DNAss, immune and stromal score in a correlation analysis between the immune microenvironment, tumor stem cells, and the risk score.

We discovered that higher expression of several prognostic genes was linked to greater medication resistance against a variety of FDA-authorized chemotherapeutic medicines, including bosutinib, lapatinib, tamoxifen, IPI145, and idelalisib, using data from NCI-60 cell lines. Of course, some prognostic genes have been connected with higher drug sensitivity for a variety of medications. For instance, increased expression of CD69, BTG2, MMP14, PCDH7, GPC3, and NMUR1 was linked to greater drug sensitivity of cancer cells to drugs such as oxaliplatin, vemurafenib, trametinib, paclitaxel, vinblastine, etc. The MRP family consists of 13 members, with MRP1–MRP9 being the primary transporters implicated in multidrug resistance *via* extruding anticancer medicines from tumor cells (Sodani et al., 2012). Therefore, the relationship between medication resistance genes including MRP1 and risk score indicated that targeting tumor medication resistance genes might be a potential therapeutic option for high-risk patients, while MRP3 is the opposite. These findings established that various prognostic genes might be employed therapeutically as targets for overcoming adjuvant drug sensitivity or drug resistance.

## Conclusion

To summarize, our research identified an 8-gene inflammatory response signature as a novel predictive factor. In both the TCGC and GEO validation cohorts, the signature was identified as being independently linked with OS, and it was also found to be useful in treatment sensitivity, functional analysis, and TME, and providing understanding in forecasting the prognosis of LUAD. The exact process linking inflammatory response-related genes to tumor immunity in LUAD is unknown, and more research is needed. Our research will go an extra mile toward elucidating their function in carcinogenesis, especially in the fields of drug resistance, TME, and immune response, which is critical for developing individualized cancer therapeutics.

## Data Availability

Publicly available datasets were analyzed in this study. This data can be found here: https://www.cancer.gov/tcga http://www.ncbi.nlm.nih.gov/geo/. GSE68465.
